# Factors Associated with Higher Reported Pain Levels in Patients with Chronic Musculoskeletal Pain: A Cross-Sectional, Correlational Analysis

**DOI:** 10.1371/journal.pone.0163132

**Published:** 2016-09-16

**Authors:** Sang Jun Park, Duck Mi Yoon, Kyung Bong Yoon, Ji Ae Moon, Shin Hyung Kim

**Affiliations:** Department of Anesthesiology and Pain Medicine, Anesthesia and Pain Research Institute, Yonsei University College of Medicine, Seoul, Republic of Korea; Associazione OASI Maria SS, ITALY

## Abstract

**Background:**

Chronic musculoskeletal pain is highly prevalent, disabling, and costly, and has many negative effects on quality of life. The aim of this study was to investigate factors associated with higher reported pain levels in patients with chronic musculoskeletal pain among demographic, clinical, and psychological factors, and to evaluate whether insomnia is independently associated with pain intensity in this population.

**Methods:**

A total of 357 patients with chronic musculoskeletal pain (pain duration ≥ six months) satisfied the study inclusion criteria and were included in the analyses. Patient demographics, clinical, and psychological factors were evaluated with hierarchical multivariate logistic analysis to identify factors associated with severe pain (NRS [numeric rating scale] ≥ 7). Hierarchical linear regression analysis also performed to identify factors associated with pain intensity (0 to 10 NRS).

**Results:**

Multivariate logistic analyses revealed older age (OR [odds ratio] = 1.017, 95% CI [confidence interval] 1.001–1.032, P = 0.034), high anxiety level (OR = 1.162, 95% CI 1.020–1.324, P = 0.024), high pain catastrophizing (OR = 1.043, 95% CI 1.007–1.081, P = 0.018), and severe insomnia (OR = 1.112, 95% CI 1.057–1.170, P<0.001) were significantly associated with severe pain. Hierarchical linear regression analysis showed age (*β* = 0.106, P = 0.041), pain catastrophizing (*β* = 0.249, P<0.001), and insomnia (*β* = 0.286, P<0.001) were significantly associated with pain intensity. The variance in pain intensity explained by the final model was 32.2%.

**Conclusions:**

Older age, severe insomnia, and high pain catastrophizing were significantly associated with higher reported pain levels. Insomnia was independently associated with pain intensity, even after controlling for various demographic and clinical factors. These factors should be considered when devising pain management strategies for this population.

## Introduction

Chronic musculoskeletal pain is highly prevalent, disabling, and costly, and has many negative effects on quality of life [1,2]. Patients with chronic musculoskeletal pain report different levels of pain with different analgesic needs, even among those with similar radiological or pathological conditions, because pain is not only a primitive sensory signal for damaged tissue, but also a complex psychological experience. Chronic pain, by its very nature, is closely related to psychological states such as insomnia, anxiety, depression, and pain catastrophizing [2–4]. These psychological states can either exacerbate or inhibit nociception and the experience of pain [3,4].

Many patients suffering from chronic musculoskeletal pain frequently experience sleep problems even when taking analgesics [5,6]. Because insomnia has been shown to worsen pain, mood, and physical functioning, it could negatively impact the clinical outcomes of patients with chronic musculoskeletal pain [7]. Insomnia is reported to be significantly associated with increased pain intensity and lowered pain threshold [8,9]. Also, insomnia is commonly seen in depressive/ anxious patients. Depression and anxiety are significantly associated with increased pain intensity [3,4,8]. On the other hand, comorbid insomnia and high pain catastrophizing could aggravate central sensitization which increases clinical pain [10]. High pain catastrophizing and heightened depressed mood showed an additive and adverse effect on the impact of pain relative to either alone [3]. Furthermore, cognitive processes such as pain catastrophizing and cognitive pre-sleep arousal seem to be related to insomnia severity in chronic pain [11]. Collectively, although the interaction between depression, anxiety, pain catastrophizing, and insomnia is complex and not fully understood, insomnia seems to significantly influence pain perception in chronic pain patients, directly or indirectly. Furthermore, a recent study demonstrated that improvement in insomnia as a moderator can lead to better treatment outcomes in chronic pain patients [12]. We hypothesized that insomnia would contribute significantly to pain perception in chronic musculoskeletal pain patients among these psychological factors.

The aim of this study was to investigate factors associated with higher reported pain levels in patients with chronic musculoskeletal pain among demographic, clinical, and psychological factors, and to evaluate whether insomnia is independently associated with pain intensity in this population.

## Methods

### Study population

This retrospective, cross-sectional study was approved by the Institutional Review Board of Severance Hospital, Yonsei University Health System (ref: 4-2016-0241). The requirement for informed patient consent was waived; however, patient records were anonymized and de-identified prior to analysis. Data was obtained from a clinical data retrieval system in our institution using musculoskeletal pain-related diagnoses. The sample population in this study comprised patients with musculoskeletal pain who received treatment for their pain and completed pain-related psychological measures between March 2015 and February 2016 at our outpatient clinic. Musculoskeletal pain was defined as pain in the following five anatomical areas: neck, shoulder or higher part of the back; elbow or wrist/hand; lower part of the back; hip or knee; and ankle and foot. Chronicity was established by the persistence of pain beyond six months. We excluded patients with current cancer and pre-existing psychiatric and neurologic disorders, which are known to affect psychological symptoms. Patients with major pathologies of the musculoskeletal structures including fractures, spinal cord injuries, infections, or neoplasms were also excluded. Patients were excluded if they reported that primary insomnia or depressive/anxious symptoms predated the onset of pain, or were diagnosed with obstructive sleep apnea or peripheral neuropathy.

### Demographic and clinical data measures

Patient data was collected, including age, gender, body mass index (BMI), main pain sites, duration of pain, pain score measured on a 0 to 10 numeric rating scale (NRS) (patients were asked to evaluate the current worst pain), current medications, presence of medical comorbidities (diagnosed hypertension, diabetes mellitus, heart diseases or neurologic diseases currently requiring medical treatment), current pain sites related to surgical history, presence of symptoms suggesting neuropathic pain (radiating pain and/or symptoms including dysesthesia or allodynia, burning or coldness, “pins and needles” sensations, numbness and itching), and presence of multisite pain (pain in two or more anatomical areas other than main pain sites).

### Psychological data measures

The severity of insomnia was assessed by the seven-item Insomnia Severity Index (ISI) [13]. The usual recall period is the “last month” and the dimensions evaluated are: severity of sleep onset, sleep maintenance, and early morning awakening problems, sleep dissatisfaction, interference of sleep difficulties with daytime functioning, noticeability of sleep problems by others, and distress caused by the sleep difficulties [13,14]. Each item is graded on a five-point scale (0 = not at all to 4 = very severe) such that the global score ranges from 0 to 28, with higher scores indicating more severe insomnia. According to the recommended score interpretation guidelines, an ISI score of 15 or more is considered indicative of clinically significant insomnia [14]. The level of anxiety and depression was assessed using the 14-item Hospital Anxiety and Depression Scale (HADS) [15]. These 14 items, each scored on a 0 to 3 scale, are used to measure the degree of anxiety (seven items) and depression (seven items). The two subscale scores range from 0 to 21, with higher scores indicating an increased likelihood of an anxiety or depressive disorder. The level of pain catastrophizing was assessed using the Pain Catastrophizing Scale (PCS) [16]. The PCS is comprised of 13 items measuring catastrophizing thoughts or feelings accompanying the experience of pain. Respondents are asked to reflect on past painful experiences and to indicate the degree to which each of the 13 thoughts or feeling are experienced when in pain. Each item is graded on a five-point scale (0 = not at all to 4 = all the time), such that the global score ranges from 0 to 52, with higher scores indicating greater pain catastrophizing. To meet the objective of this study, the ISI, HADS, and PCS measurements were based on review of memory within a month prior to the pain assessment time in individuals. These psychological measures were performed by independent resident doctors during the preliminary medical examination.

### Statistical analysis

Continuous variables were recorded as means ± SDs, and categorical variables as a number (percentage). The outcome variable in this study was the worst level of pain during last two weeks either assessed as a dichotomous variable (pain severity: NRS <7 or ≥ 7) or as a continuous variable (pain intensity: NRS 0 to 10). For the dichotomous outcome, patients were classified into 2 groups, mild to moderate pain (NRS < 7 for ‘‘worst pain level”) and severe pain (NRS ≥ 7 for ‘‘worst pain level”). We selected this cut-point with NRS ≥ 7, which would be reflected more hyperalgesic state. Also, mean pain score of 481 chronic back pain patients in our clinic was 6.6 in our previous study. [17].

Demographic, clinical, and psychological measures were compared between patients with mild to moderate pain (NRS<7) and severe pain (NRS ≥ 7) using a t-test or chi-square test as univariate analyses. Significant variables (*P* <0.05) on univariate analysis were selected for multivariate analysis. In addition, Pearson correlation coefficients were also calculated among study variables. Hierarchical multivariate logistic regression was used to compute adjusted odds ratios (ORs) with 95% confidence intervals (CIs) for predictors associated with severe pain (NRS ≥ 7). Demographic, clinical, and psychological measures (anxiety, depression, and pain catastrophizing) were sequentially entered in Step 1 to 3. ISI data for insomnia was finally entered in the final model (Step 4). Hierarchical linear regression was performed to identify significant predictors of pain intensity as the outcome. Measures were entered in Step 1 to 4 in the same order as for the logistic regression. To control for the influence of multicollinearity, the variance inflation factor value for every independent variable was examined. The variable was included if this value was < 3. The option to use both logistic and linear regression to investigate predictors of a high level of pain was related to our interest in both pain severity and intensity as outcome variables and the thought that replication of findings via both logistic and linear regression would reinforce their robustness. Statistical analysis was performed with the Statistical Package for the Social Sciences (SPSS, version 20.0; SPSS Inc., Chicago, IL, USA). A *P* value of less than 0.05 was considered statistically significant.

## Results

**Fig 1:**
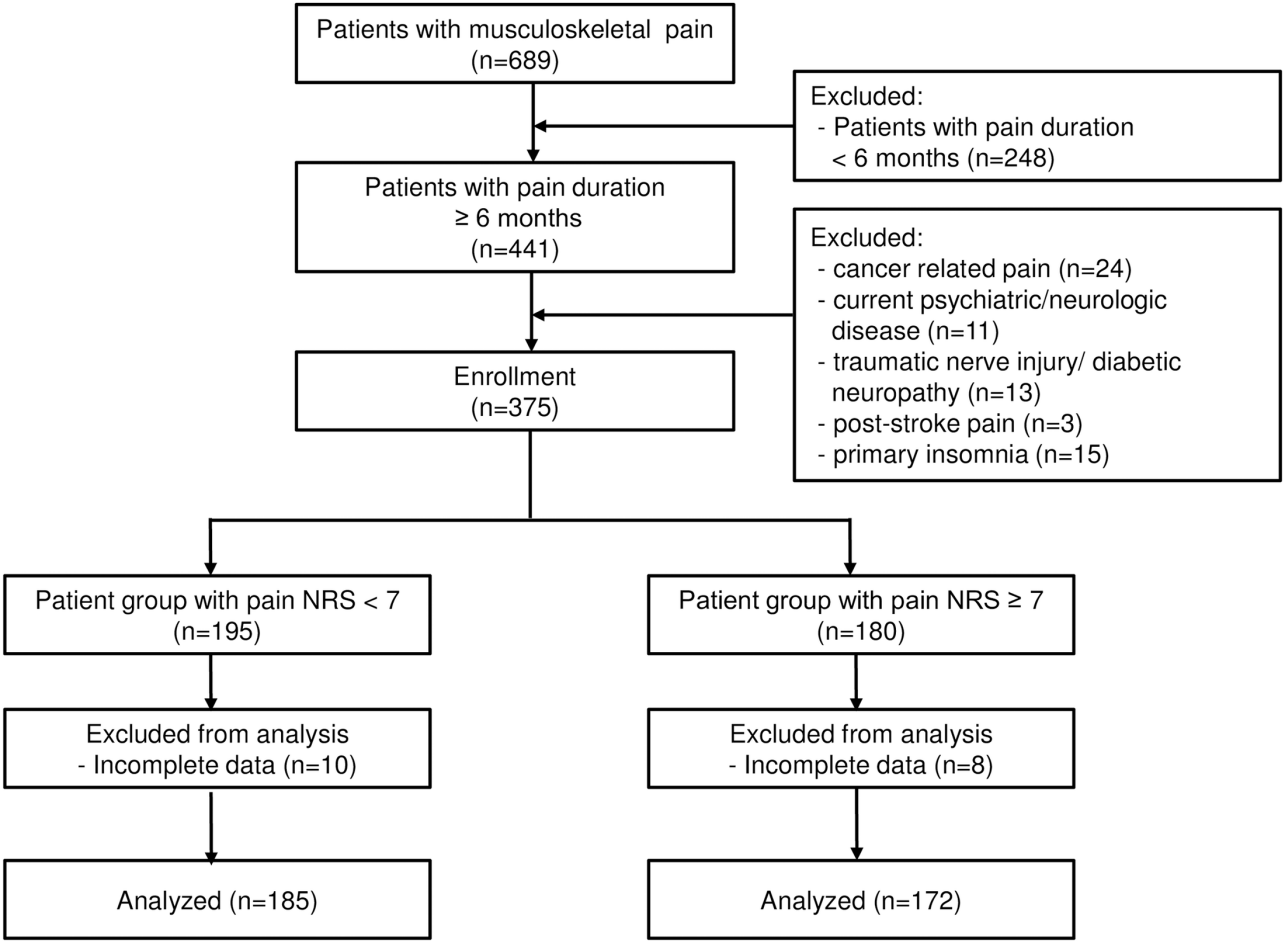
Flow diagram of the study. NRS = numeric rating scale.

Data from 689 patients who were treated for musculoskeletal pain as their chief complaint at our pain clinic were obtained from the electronic medical records. After excluding cases with pain duration < six months, a total of 441 patients were evaluated for eligibility. Twenty-four patients with cancer-related pain and eleven patients with pre-existing major psychiatric and neurologic disorders such as major depressive disorder and dementia were excluded. Seven patients with traumatic peripheral nerve injury and three patients with post-stroke pain syndrome were excluded. Six patients taking medication for diabetes mellitus neuropathy were excluded. Fifteen patients were excluded for having primary insomnia or depressive/anxious symptoms that emerged prior to the onset of pain. Eighteen patients with incomplete data also were excluded. Finally, a total of 357 patients with chronic musculoskeletal pain satisfied the study inclusion criteria and were included in the analyses (Fig 1).

Most patients suffered from neck and low back pain (67.5%) and joint pain (22.3%). All enrolled patients were taking more than one type of analgesic medication; 57% of patients were taking more than two types of analgesic medications. A total of 38% of patients were undergoing physical therapy or injection therapy. The mean global ISI score was 8.6 and 47.6% of patients reported mild to severe insomnia symptoms (ISI score ≥8) after pain development. Moderate to severe clinical insomnia (ISI score ≥15) was observed in 19.3% of the study population. Demographic and clinical characteristics and psychological measures of the total patient sample and in each pain severity group (NRS <7 and ≥ 7) are listed in Table 1. The mean age of the severe pain group was higher than that of the mild-moderate pain group (*t* = -3.499, P = 0.001). Female patients reported more severe pain than male patients, but the difference was not statistically significant (χ^2^ = 3.106, P = 0.082). Patients with pain site-related surgery reported more severe pain compared to those without an operative history (χ^2^ = 7.610, P = 0.006). In addition, patients with severe pain showed poor psychological states, with more severe anxiety (*t* = -8.728, P<0.001), depression (*t* = -6.529, P<0.001), pain catastrophizing (*t* = -8.970, P<0.001), and insomnia (*t* = -8.251, P<0.001).

**Table 1:** Differences between patient groups with mild to moderate pain (NRS<7) and severe pain (NRS ≥ 7) in demographic and clinical characteristics, and psychological measures.

Patients characteristics	Total sample (n = 357)	Mild to moderate pain (n = 185)	Severe pain (n = 172)	p-value
***Demographic data***				
Female/ male, n	219 (61.3%)/138 (38.7%)	105 (56.7%)/80 (43.3%)	114 (66.2%)/ 58(33.8%)	0.082
Age, years	56.1 ± 17.3	53.1 ± 18.3	59.3 ± 15.5	0.001
Body mass index, kg/m^2^	23.6 ± 3.3	23.5 ± 3.1	23.6 ± 3.2	0.861
***Clinical data***				
Pain duration, months	34.2 ± 50.0	31.5 ± 51.6	37.1 ± 48.2	0.291
Medical comorbidities (Yes/no), n	135 (37.8%)/222 (62.2%)	67 (36.2%)/118 (63.8%)	68 (39.5%)/ 104(60.5%)	0.585
Surgery history (Yes/no), n	74 (20.7%)/283 (79.3%)	28 (15.1%)/157 (84.9%)	46 (26.7%)/ 126(73.3%)	0.006
Neuropathic pain components (Yes/no), n	236 (66.1%)/121(33.9%)	112 (60.5%)/73(39.5%)	115 (66.8%)/57(33.2%)	0.911
Multisite pain (Yes/no), n	54 (15.1%)/303(84.9%)	23 (12.4%)/162(87.6%)	30 (17.4%)/142(82.6%)	0.233
***Psychological measures (continuous variables)***				
Insomnia (ISI)	8.6 ± 5.7	6.4 ± 4.5	10.9 ± 5.9	<0.001
Anxiety (HADS)	5.4 ± 3.6	4.0 ± 2.7	7.0 ± 3.8	<0.001
Depression (HADS)	4.1 ± 3.3	3.0 ± 2.7	5.2 ± 3.2	<0.001
Pain catastrophizing (PCS)	16.3 ± 10.6	11.9 ± 8.2	21.1 ± 10.9	<0.001

Values are expressed as the mean ± SD or number of patients (%). NRS = numeric rating scale; ISI = Insomnia Severity Index; HADS = Hospital Anxiety and Depression Scale; PCS = Pain Catastrophizing Scale

The results of multivariate logistic regression are presented in Table 2. The variables that emerged as predictors of severe pain (NRS ≥ 7) in the final model were age (OR = 1.017, 95% CI 1.001–1.032, P = 0.034), anxiety (OR = 1.162, 95% CI 1.020–1.324, P = 0.024), pain catastrophizing (OR = 1.043, 95% CI 1.007–1.081, P = 0.018), and insomnia (OR = 1.112, 95% CI 1.057–1.170, P<0.001), with older patients and those with an increased level of anxiety, pain catastrophizing, and insomnia having a higher probability of being in the severe pain group. Female gender and level of depression were not significant factors in this analysis. Surgical history was a significant factor in Step 2, but it was no longer significant after psychological measures were entered in Steps 3 and 4.

**Table 2:** Hierarchical multivariate logistic regression analysis for risk factors predicting severe pain (NRS ≥ 7, n = 172) in 357 chronic musculoskeletal pain patients.

Variables	Odds ratio (95% CI)	p-value
***Step 1***		
Female gender	1.332 (0.858–2.067)	0.202
Age	1.021 (1.008–1.034)	0.001
***Step 2***		
Surgery history	1.919 (1.119–3.292)	0.018
***Step 3***		
Anxiety	1.195 (1.050–1.359)	0.007
Depression	0.986 (0.878–1.108)	0.815
Pain catastrophizing	1.054 (1.019–1.090)	0.003
***Step 4 (final model)***		
Female gender	1.142 (0.663–1.909)	0.613
Age	1.017 (1.001–1.032)	0.034
Surgery history	1.724 (0.907–3.276)	0.097
Anxiety	1.162 (1.020–1.324)	0.024
Depression	0.964 (0.855–1.087)	0.549
Pain catastrophizing	1.043 (1.007–1.081)	0.018
Insomnia	1.112 (1.057–1.170)	<0.001

CI = confidence interval; NRS = numeric rating scale.

Pearson correlation coefficients between pain intensity and other study variables (continuous variables) were calculated. Pain intensity was significantly correlated with age (r = 0.128, p = 0.001) and psychological measures such as depression (r = 0.350, p<0.001), anxiety (r = 0.435, p<0.001), pain catastrophizing (r = 0.478, p<0.001), and insomnia (r = 0.462, p<0.001). Results of variance inflation factor analysis showed that there was no problematic multicollinearity among the psychological measures (1.350 to 2.334). Hierarchical linear regression analysis was performed to identify predictors of pain intensity (Table 3). As mentioned in the methods section, this model was generated using the same sequential steps as previously described for pain severity. Three psychological measures, anxiety, depression, and pain catastrophizing were included in step 3, and anxiety (*β* = 0.177, P = 0.042) and pain catastrophizing (*β* = 0.323, P<0.001) were found to be significant predictors. The variance explained by the first two steps was only 5.0%, whereas the variance explained by step 3 (anxiety, depression, and pain catastrophizing entered) remarkably increased to 26.1%. Insomnia was entered in the final step, and also emerged as a significant predictor (*β* = 0.286, P<0.001), explaining an additional 6.0% of the variance in pain intensity. Finally, the variance explained by the final model increased to 32.2%. Anxiety (*β* = 0.113, P = 0.211) was not a significant predictor in the final model.

**Table 3:** Hierarchical linear regression analysis for predictors of pain intensity (0 to 10 NRS) in 357 chronic musculoskeletal pain patients.

Variables	*t*	Standardized *β*	*R*^*2*^	*R*^*2*^ change	*F* change
***Step 1***			0.034	0.034	6.185**
Female gender	0.863	0.046			
Age	3.238**	0.171			
***Step 2***			0.050	0.016	6.108*
Surgery history	2.472*	0.130			
***Step 3***			0.261	0.211	33.352***
Anxiety	2.037*	0.177			
Depression	0.092	0.007			
Pain catastrophizing	4.562***	0.323			
***Step 4 (final model)***			0.322	0.060	30.934***
Female gender	-0.159	-0.007			
Age	2.335*	0.106			
Surgery history	1.309	0.061			
Anxiety	1.333	0.113			
Depression	-0.276	-0.019			
Pain catastrophizing	3.593***	0.249			
Insomnia	5.562***	0.286			

NRS = numeric rating scale.

* P<0.05,

** P<0.01,

*** P<0.001

## Discussion

In the present study, we confirmed that poor psychological states are prevalent in chronic musculoskeletal pain patients with severe pain. We found that older age, high pain catastrophizing, and severe insomnia were strongly associated with higher reported pain levels in this population. Insomnia was independently associated with pain intensity, even after controlling for various demographic and clinical factors.

Many studies have reported high prevalence of anxiety, depression, and pain catastrophizing together with insomnia in patients with chronic pain, and these symptoms, even though they are distinct processes, may share common neurophysiological mechanisms and behavioral manifestations [18,19]. Our results demonstrated that insomnia and pain catastrophizing significantly affects patient reported pain severity and intensity in chronic musculoskeletal pain.

Insomnia is frequently experienced by patients suffering from chronic musculoskeletal pain [15], but is often seen as a secondary symptom of chronic pain and not as an independent symptom. Because pain mediates sleep problems, adequate pain management was thought to lead to improved sleep in patients with chronic pain [20,21]. However, this point of view has shifted gradually as new evidence has emerged pointing toward insomnia as the primary disorder from which chronic pain often develops [22–24]. Interestingly, both pain-related insomnia and primary insomnia have large similarities to sleep patterns and psychological characteristics [25]. Many patients with chronic pain continue to experience sleep problems even when good pain control is achieved [26,27]. Furthermore, a recent study demonstrated improvement in insomnia resulted in better pain treatment outcomes in patients with chronic pain and comorbid insomnia [12]. From a biophysiologic perspective, insomnia has a harmful impact on carbohydrate metabolism and endocrine function [28], and is also related to increased production of inflammatory mediators, which can potentially aggravate pain [29]. Specifically, insomnia causes a hyperalgesic status with a low pain threshold, and this effect remains even after controlling other psychological symptoms [8,30]. A recent study demonstrated this insomnia-induced low pain threshold could consequently lead to an inability to sufficiently activate pain inhibitory pathways [31]. Moreover, in patients with major depressive disorder, insomnia remained a significant predictor of pain even after controlling for the severity of anxiety and depression [23]. Therefore, although the clear direction of the relationship between insomnia and pain could not be established in this study, our results indicate that specific assessment and treatment of insomnia may be emphasized in the course of pain management in patients suffering from severe chronic musculoskeletal pain.

In this study, the results of both multivariate analyses showed that pain catastrophizing was strongly associated with higher reported pain levels in patients with chronic musculoskeletal pain. This result indicates that cognitive processes related to pain may play an important role in pain perception in this population. Catastrophizing has been defined as an exaggerated negative mindset brought to bear during actual or anticipated painful experiences [32]. It is a key factor as to how cognition, beliefs, coping strategies and functioning are related to the experience of pain [33]. Although few studies address the role of pain catastrophizing in the relationship between insomnia and pain, a significant portion of variance in clinical pain severity is attributable to pain catastrophizing, and rumination about pain in particular was mediated by sleep disturbances in a population suffering from chronic myofascial pain [34]. Patients with osteoarthritis, comorbid insomnia and a high level of catastrophizing reported increased clinical pain following increased levels of central sensitization [10]. Collectively, pain catastrophizing should be considered in the sleep-pain relationship, although the underlying cognitive processes in comorbid insomnia and chronic pain are not yet clear [11].

An interesting finding was that chronological age was significantly associated with patient-reported pain severity and intensity in this study. In experimental studies, low pain threshold, the temporal summation of low-frequency stimuli, and capsaicin-induced persistent secondary hyperalgesia were more frequently observed in older subjects when compared with younger subjects [35–37]. This indicates that the reduced efficacy of endogenous analgesic systems, a decreased tolerance of pain and the slower resolution of post-injury hyperalgesia may make it more difficult for the older adult to cope, once injury has occurred [38]. These age-related changes of pain perception may be intensified by the burden of comorbidities and psychological symptoms including insomnia in older individuals [39]. Worse psychological states were also observed as age increased in this study; therefore, the severity of comorbid psychological symptoms seems to be important when devising treatment strategies for older patients who are likely to be especially vulnerable to the negative impacts of pain and pain-associated events.

Our study has several limitations that suggest directions for future research. First, it was conducted in a single clinical setting and included a study population with a homogeneous racial background, which might limit our ability to detect potentially significant associations. Secondly, our findings are cross-sectional in nature, limiting our ability to identify a causal relationship between psychological factors and current pain or treatments. We also could not control for potential confounders such as anticonvulsant and antidepressant use, which could affect sleep or mood. Third, pain intensity as an outcome variable was only measured by patient-reported pain score using NRS, and this study was based on subjective assessment of insomnia. Fourth, the ISI does not include parameters that are used to measure the degree of interruption in daytime activities, and insomnia frequency and duration, which would influence insomnia severity. Therefore, prospective and longitudinal studies with clear temporal precedence and causality should be needed to evaluate the influence of psychological symptoms including insomnia on pain intensity in chronic musculoskeletal pain.

In conclusion, we found that older age, severe insomnia, and high pain catastrophizing were significantly associated with higher reported pain levels in chronic musculoskeletal pain patients. Therefore, these factors should be addressed as an important part of pain management in this population. Especially, specific assessment and treatment of insomnia should be emphasized in the course of pain management in patients with severe pain.

## Supporting Information

S1 Dataset (XLSX)
